# Preferences for a potential longer-acting injectable contraceptive: perspectives from women, providers, and policy makers in Kenya and Rwanda

**DOI:** 10.9745/GHSP-D-13-00147

**Published:** 2014-03-26

**Authors:** Elizabeth E Tolley, Kevin McKenna, Caroline Mackenzie, Fidele Ngabo, Emmanuel Munyambanza, Jennet Arcara, Kate H Rademacher, Anja Lendvay

**Affiliations:** a FHI 360, Durham, NC, USA; b FHI 360, Nairobi, Kenya; c Ministry of Health, Maternal and Child Health Department, Kigali, Rwanda; d FHI 360, Kigali, Rwanda; e University of North Carolina-Chapel Hill, Chapel Hill, NC, USA

## Abstract

**Background:**

Between 1995 and 2005, injectable use doubled worldwide. However, discontinuation rates remain high, partly because of side effects but also because of missed appointments for reinjection. A longer-acting injectable (LAI) may improve compliance by reducing the required number of reinjection visits, thereby reducing unintentional discontinuation. This study examined acceptability of LAI characteristics comprising the target product profile (TPP).

**Methods:**

In 2012, we conducted qualitative case studies in Kenya and Rwanda, consisting of 19 focus group discussions (FGDs) with 177 current, previous, or never users of injectables and 46 in-depth interviews (IDIs) with providers, program implementers, and policy makers. FGDs and IDIs assessed current injectable experiences; attitudes toward potential LAI products; and perceptions of TPP attributes, including ranking preferences for the most and least important characteristics. In addition, we obtained completed electronic surveys from 28 international family planning opinion leaders about the perceived need for an LAI, important product characteristics, and challenges to LAI development or introduction.

**Results:**

Many FGD participants and interviewees spontaneously expressed strong interest in an LAI, but there was some variation in TPP preferences. The majority of participants ranked effectiveness as the most important TPP attribute. Providers were generally more concerned about side effects than potential users; some potential users suggested that side effects were related less to the product than to their own body chemistry and that side effects were acceptable as long as they did not last a long time or disrupt daily activities. Women and providers, especially in Kenya, preferred a method with a predictable return to fertility. Some participants associated amenorrhea with delayed or reduced fertility. Most women and providers preferred delivery of the LAI in a single, prepackaged, disposable injection system to facilitate injections by providers and to reduce the risk of pain or discomfort for women. While providers and policy makers ranked cost as one of the most important issues, it was among the least important issues for most potential users. Many Kenyan, but few Rwandan, participants appeared willing to pay for an LAI, with some presuming cost savings from reduced menstruation and fewer clinic visits.

**Conclusions:**

Some TPP preferences for an LAI have implications for product development decisions about formulation, delivery mechanism, or presentation, while others point to the need for tailored communication and counseling approaches to ensure acceptability and adherence within clinical trials and beyond.

## Abstract (precis)

High effectiveness, predictable return to fertility, and a single, prepackaged, disposable delivery system ranked high. Side effects were generally acceptable to women if they did not last long or disrupt daily activities. Cost was considered important for providers but not so much for most potential users.

## BACKGROUND

Currently, more than 40 million women use injectable contraceptives to prevent pregnancy.^1^ The most common injectable contraceptive, depot medroxyprogesterone acetate (DMPA), is a progesterone-only product administered through a single intramuscular injection in the upper arm or buttocks that lasts for 3 months. Other injectable contraceptives include 1-month combined hormonal products and a 2-month progesterone product.^2^

Between 1995 and 2005, injectable contraceptive use doubled worldwide.^3^ In sub-Saharan Africa, more than one-third of modern contraceptive users currently rely on injectable contraceptives.^4^ Dramatic increases in injectable uptake have occurred in several African countries, including Kenya and Rwanda.^5^^,^^6^ However, injectable discontinuation is high, due in part to women experiencing menstrual and weight changes or other side effects.^7^^–^^12^ Additionally, as many as 40% of injectable users unintentionally discontinue due to missed appointments for reinjection.^13^^,^^14^ Injectable users who are late for a reinjection are often asked to return to the clinic during their next menses, increasing the likelihood of pregnancy before the next injection.^15^

Between 1995 and 2005, injectable use doubled worldwide, but discontinuation remains high due to side effects and missed reinjections.

A longer-acting injectable (LAI) lasting for at least 6 months could prove to be a valuable addition to the method mix by decreasing the number of visits required of clients per year, thereby improving compliance and increasing effectiveness during typical use of the method. With funding from the Bill & Melinda Gates Foundation, FHI 360 is spearheading efforts to develop an LAI that would provide protection for a minimum of 6 months. Approaches under consideration include: (a) increasing the dosage of an existing injectable formulation, (b) altering the administration or injection site, or (c) identifying drug delivery systems that could prolong the release of the drug.

By reducing the number of reinjection visits, a longer-acting injectable could potentially improve compliance and reduce unintentional discontinuation.

This research aimed to inform the development process of an LAI by providing a more in-depth understanding of the perspectives of potential users, providers, and opinion leaders on the target product profile (TPP), which identifies desired and minimally acceptable product characteristics related to such aspects as effectiveness and side effect profile. The results are intended to help inform the selection of potential product formulations for early proof-of-concept testing as well as for later product development efforts. Another paper is planned to present and discuss how TPP characteristics may affect health systems and introduction decisions more broadly. Such decisions will influence women's access to an LAI by affecting the method's affordability, availability, and ease of adoption, further affecting how well potential users can adhere to and sustain use of the new method.

## METHODS

Between June and September 2012, we conducted qualitative case studies in Kenya and Rwanda, 2 countries with high levels of injectable contraceptive use but different service delivery environments. In each country, we recruited providers, including nurses, counselors, and community health workers (CHWs) as well as potential users from private- and public-sector clinics in the capital city and several peri-urban or rural areas.

Research assistants approached women in clinic waiting rooms to inform them about the study. The research assistants screened interested women to ensure they were 18–50 years old, and they provided women with an informational voucher to attend a focus group discussion (FGD) with current and previous injectable users or with women who had never used injectables.

Policy makers represented the Ministry of Health, public sector, and private sector/nongovernmental organizations (NGOs).

In total, we conducted 19 FGDs with 177 women (Table 1) as well as in-depth interviews (IDIs) with 27 service providers (15 in Kenya and 12 in Rwanda) and 19 policy makers and program implementers (12 in Kenya and 7 in Rwanda). FGDs were conducted in the local language by trained female interviewers following a topic guide to explore potential users' DMPA-related knowledge and experiences; to discuss new LAI approaches; and to explore LAI characteristics identified in the TPP. IDIs were conducted by male or female interviewers in English, French, or the local language, following a similar guide.

**TABLE 1.** Composition of Focus Group Discussions Among Potential Users of a Longer-Acting Injectable, by Country

**FGD Number**	**Location**	**Facility Type**	**Experience With Injectable Use**	**No. of Participants**	**Mean Age (Range)**
**Kenya**					
1	Urban	Public health center	Current and past users	10	28.1 (21–38)
2	Urban	Public health center	Never users	8	36.7 (18–45)
3	Peri-urban	Public hospital	Current and past users	11	27.7 (21–37)
4	Peri-urban	Public health center	Current and past users	8	25.2 (20–32)
5	Urban	NGO health center	Current and past users	10	31.0 (23–40)
6	Urban	NGO health center	Current and past users	8	29.4 (25–37)
7	Urban	NGO health center	Never users	9	30.3 (23–36)
8	Peri-urban	Public health center	Current and past users	10	29.9 (22–40)
9	Peri-urban	Public health center	Current and past users	11	29.4 (25–38)
10	Peri-urban	Public hospital	Never users	8	31.6 (20–42)
**Total**				**93**	
**Rwanda**					
1	Rural	Public CBD	Current, past, and never users	10	32.4 (24–50)
2	Rural	Public health center	Current and past users	7	30.7 (26–39)
3	Peri-urban	Public health center	Never users	13	28.0 (20–39)
4	Urban	Public hospital	Never and past users	11	28.4 (21–37)
5	Rural	Public CBD	Current users	8	37.7 (28–44)
6	Rural	Public health center	Current and past users	9	31.3 (21–40)
7	Rural	Public CBD	Current and past users	9	34.8 (21–44)
8	Urban	Public hospital	Current users	7	28.6 (22–35)
9	Urban	NGO health center	Current, past, and never users	10	31.9 (25–44)
**Total**				**84**	

Abbreviation: CBD, community-based distribution.

During the FGDs and IDIs, interviewers used illustrations depicting each TPP characteristic to facilitate discussion. At the end of each FGD or IDI, the interviewers presented the illustrations to the participants again and asked them to prioritize the 3 most important and 3 least important product characteristics in the development of a new LAI. FGD participants had to reach consensus. (In 2 FGDs, participants split into 2 groups and provided 2 separate rankings.)

IDIs and FGDs were audio-recorded, translated into French or English, and transcribed. The documents were then uploaded into NVivo 9, and the information was coded and analyzed thematically. We wrote detailed memos describing subthemes related to each main code, including each of the TPP characteristics. We also created Excel matrices to examine variations in subthemes by country and participant type.

In addition to the IDIs and FGDs, we distributed an electronic survey to 95 individuals from international funding agencies, foundations, NGOs, and universities who were identified as international family planning opinion leaders by peers. Organizations included the International Planned Parenthood Federation, the United Nations Population Fund, the World Health Organization, the U.S. Agency for International Development, Abt Associates, the Population Council, Management Sciences for Health, Marie Stopes International, and others. We received 28 responses. The opinion leaders were asked open-ended questions about whether they perceived a need for an LAI; what characteristics would be important; and what challenges might exist related to LAI development and introduction. Responses were organized into a matrix by topic.

The study was approved by FHI 360's Protection of Human Subjects Committee and by the Institutional Review Boards in Kenya and Rwanda.

## RESULTS

**FIGURE 1.**
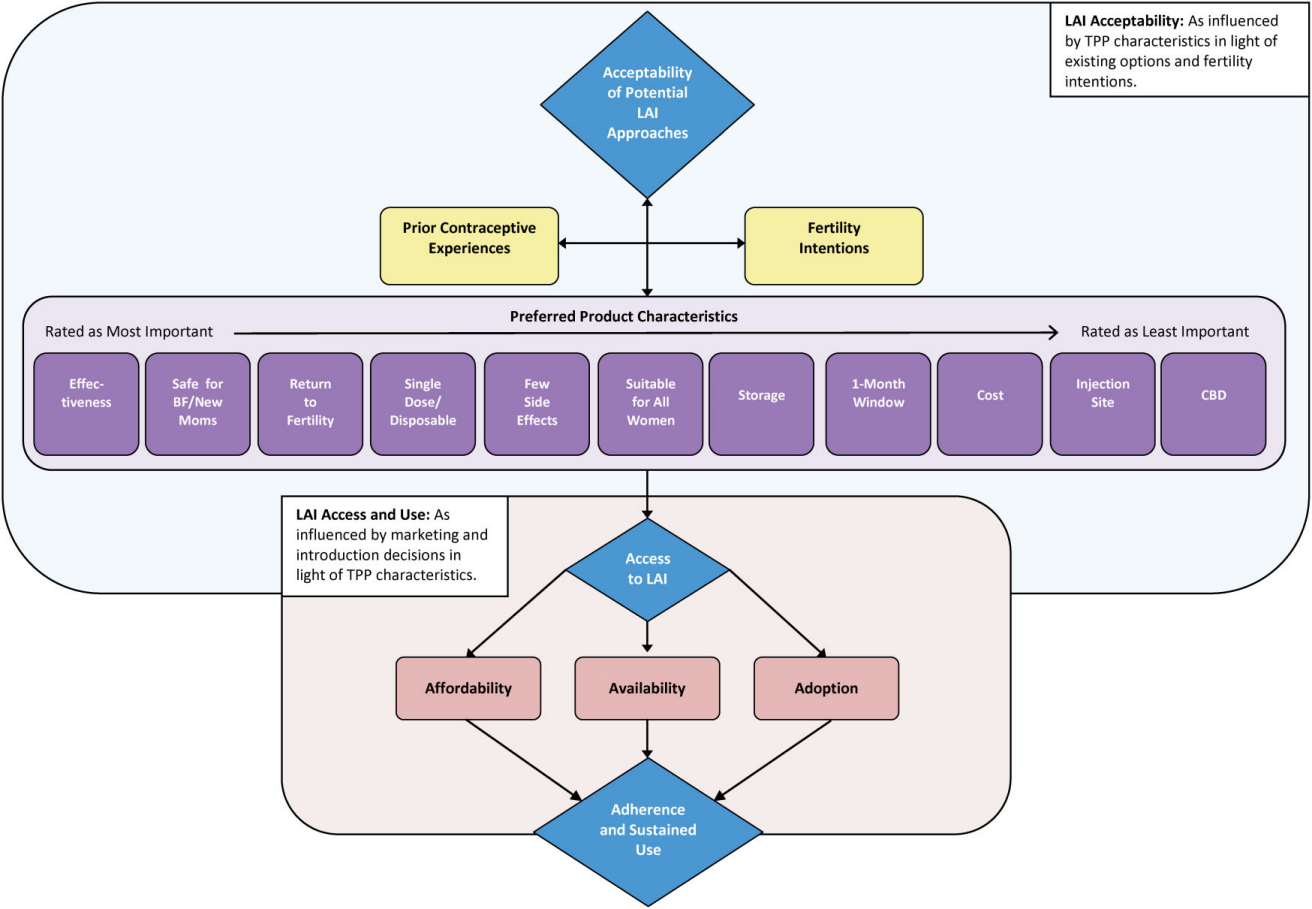
Conceptual Framework: Factors Influencing Acceptability of, Access to, and Use of a Potential Longer-Acting Injectable Abbreviations: BF, breastfeeding; CBD, community-based distribution; LAI, longer-acting injectable; TPP, target product profile.

The conceptual framework in Figure 1 provides an overview of the themes we analyzed and their relationship to LAI acceptability. Many women and providers/policy makers spontaneously expressed strong interest in an LAI. In general, we found that women's interest in an LAI was informed by specific TPP product characteristics that influenced how well the product fit their fertility desires. However, for providers, policy makers, and international opinion leaders, interest in an LAI was informed by its contribution, relative to other temporary or long-acting contraceptive methods, to a country's method mix.

In addition, participants' attitudes toward specific product attributes were often framed in terms of their experiences with using or providing other available contraceptive options. These experiences were influenced by individual, sociocultural, and health systems contexts that led to some interesting variations in attitudes and preferences by participant type (providers versus potential users) and by country.

**FIGURE 2.**
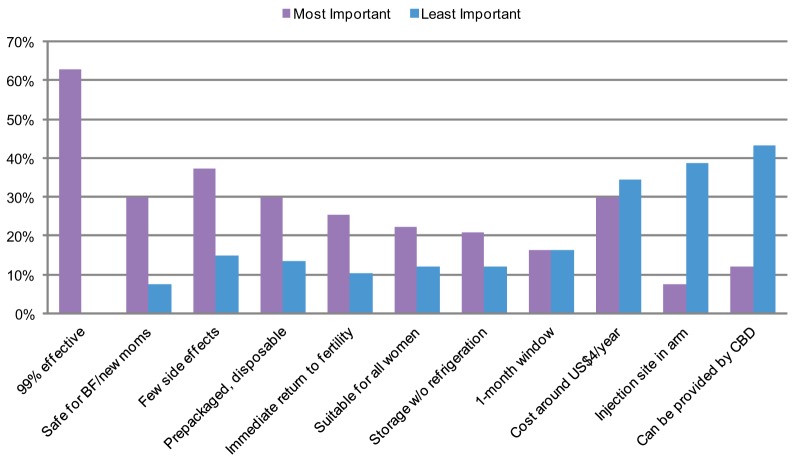
Most and Least Important Attributes of a Potential Longer-Acting Injectable, Ranked by Women, Providers, and Policy Makers in Kenya and Rwanda (N = 67)^a^ Abbreviations: BF, breastfeeding; CBD, community-based distribution. ^a^ Rankings are among 46 providers and policy makers from in-depth interviews and 21 groups of women (from 19 focus groups with 2 focus groups further split into 2 groups for their rankings) for a total sample size of 67.

Figure 2 presents the most and least important product attributes of a new LAI ranked by the participants, while Table 2 shows the 3 most and 3 least endorsed attributes by country and participant type. (Rankings of providers and policy makers were counted individually while rankings of women were counted per focus group.) The majority of participants ranked effectiveness as one of the most important characteristics of a new injectable. However, there was less agreement on many other characteristics.

**TABLE 2.** Three Most and Least Important Attributes of a Potential Longer-Acting Injectable, by Participant Type and Country^a^

**Attributes**	**Most Important**						**Least Important**					
	**Potential Users**		**Providers**		**Policy Makers**		**Potential Users**		**Providers**		**Policy Makers**	
	**Kenya**	**Rwanda**	**Kenya**	**Rwanda**	**Kenya**	**Rwanda**	**Kenya**	**Rwanda**	**Kenya**	**Rwanda**	**Kenya**	**Rwanda**
99% effective	1	2	1	2	1	1						
Safe for BF and new mothers	3	1		2								
Immediate return to fertility	2		2		3			2				2
Storage without refrigeration				1							3	
Prepackaged, single dose, disposable	3	2				2						
Cost around US$4/year			3	2		2	2	1			3	
Side effects no worse than current methods	3		3	3	2			3				
Suitable for all women		3		3	3	3	3					
6-month duration with 1-month window									3	2	2	3
Can be administered in arm							3	3	1	3	1	1
Can be provided by CBD							1	1	2	1		

Abbreviations: BF, breastfeeding; CBD, community-based distribution.

a In Kenya, rankings are among 10 groups of potential users, 15 providers, and 12 policy makers. In Rwanda, rankings are among 9 groups of potential users, 12 providers, and 7 policy makers.

In this paper, we describe in detail participants' perspectives on several TPP characteristics with high, low, or variable consensus across participant groups. For each selected characteristic and TPP description, we first describe how past contraceptive experiences shape participants' assessment of its importance and then transition to how the characteristic affects hypothetical LAI acceptability. We also indicate when such perspectives diverge between potential users and providers or by country, and how they compare to responses from the international opinion leaders.

### Effectiveness: 99% Effective in Preventing Pregnancy When Used Correctly

Being at least 99% effective was ranked as the most important LAI characteristic by most participant groups from the 2 country case studies, but mentioned by only 7 of 28 international opinion leaders as a critical characteristic, probably because they assumed high effectiveness was a given. As one opinion leader noted, “Safety and efficacy are of course a no-brainer.”

High effectiveness was ranked the most important characteristic of a potential longer-acting injectable by most participants.

Despite strong endorsement from Kenyan and Rwandan participants, their understanding of “effectiveness” was often based less on a quantitative understanding of rate and more on subjective experiences with other contraceptive methods. About a dozen potential users and providers incorrectly suggested that “all other contraceptive methods are 100% effective,” so an LAI should also be 100% effective. Similarly, a Kenyan provider said:

I have never heard [of] any person who has ever used Depo and still conceived when the method was used correctly.

In contrast, twice as many women believed that no method was completely effective. A Kenyan woman who had never used injectables acknowledged:

It's okay [if the method is 98% or 99% effective] because there are no methods that are 100% [effective].

A handful of past and never users set minimum effectiveness as low as 45%–50%, before other FGD participants clarified the meaning of effectiveness rates.

**DMPA and Other Contraceptive Experiences:** More generally, women and providers thought of an LAI as extending the period of protection of the existing DMPA injectable, making it even easier and more convenient to use. In both countries, potential users and providers frequently contrasted the reliability of the 3-month injectable to women's experiences using oral contraceptive pills. For example, a young Rwandan injectable user explained:

Usually, what's good about the 3-month injectable is that you are at ease for 3 months. It's only important not to forget the date to go back [to the clinic]. It's not like with pills that you can forget to take.

Despite a widespread perception that current injectable contraception was highly effective, some women in almost all Kenyan FGDs and half of Rwandan FGDs also described instances of their own or another's unplanned pregnancy while using injectables. Such instances were usually attributed to returning late for a reinjection. However, in 7 FGDs in Kenya, participants attributed contraceptive failure to the possibility of being administered a “fake” or an expired drug. This concern led one Kenyan contraceptive user to choose pills:

… because I'm assured of not getting pregnant when I'm on the pills, unlike the injection where some people are injected with water instead of the drug.

**Effectiveness and LAI Approaches:** Potential users and providers had little problem imagining how to achieve a highly effective LAI based on increasing the dose of an existing hormone. However, some participants wondered how other approaches to achieving an LAI might work. For example, a subcutaneous injection was viewed by some as likely to be less effective. One Kenyan woman from a non-injectable user group wondered:

How is the drug going to move? … Because when you are injected, they usually target a vein that is going to transport the medicine all over. So if it is under the skin, where are they aiming it? [I would prefer it to be injected] deep in the muscles, so I am sure it is going to reach the veins and the medicine will be transported all over.

A subcutaneous injection was viewed as being less effective than an intramuscular injection by some participants.

Such views emerged strongly in 2 Kenyan FGDs of non-users as well as in 1 FGD of current or past injectable users. Another Kenyan woman indicated:

Okay for less pain, I would prefer under the skin, but to be sure it will work, I would go for the deeper one.

In Rwanda, at least a few participants in FGDs of current, past, or never users also suggested that an intramuscular injection might be more effective than a subcutaneous one. However, slightly more Rwandan potential users had a clear preference for subcutaneous injections, which they believed would be less painful.

### Side Effects: No Worse Than Currently Available Hormonal Methods/Injectables

Providers, potential users, and opinion leaders had different perspectives on the relative importance for a new LAI to minimize side effects. Among all Kenyan focus groups as well as among providers in both countries, ensuring that LAI side effects are no worse than currently available methods was among the 3 most endorsed characteristics. In contrast, it was ranked as a least important characteristic among potential users in Rwanda.

Minimizing side effects was more important for potential users in Kenya than in Rwanda.

When opinion leaders were asked about important considerations for an LAI, the most frequent response, reported by 21 of 27 opinion leaders, was side effects. Some opinion leaders stated that the side effects associated with an LAI should be no worse than those associated with DMPA, while others expressed the hope that an LAI would have a better side effect profile than currently available injectables.

**DMPA Experiences:** Providers and potential user groups associated a number of side effects with current injectable use, including increased menstrual bleeding and amenorrhea, decreased libido, weight changes, headaches, and dizziness. Certain side effects—especially heavier or prolonged bleeding and decreased libido—could disrupt marital relations and/or work patterns, making them less tolerated than other side effects. The possibility of discreet contraceptive use was cited by several Kenyan policy makers and at least a dozen women in each country as an important reason to use the injectable.

Side effects, such as increased bleeding and decreased libido, that disrupt marital relations or daily activities were less tolerated than other side effects.

In Rwanda, a 38-year-old injectable user with 4 children described her symptoms:

I am using the 3-month injectable but I bleed and don't dry out. Currently, I have some serious problems with my husband because I am turning him away; we're not really on good terms. Sometimes he asks me to stop; he also asks me why I am using it.

A community health worker in Rwanda suggested:

Prolonged or heavy bleeding and also decreased sexual desire [weigh most on clients]. What is not very worrisome [for women] is amenorrhea. But weight gain is also a problem for women because the way they change shape is really noticeable and rapid. And then having no desire for your husband even though he is the one who brought you from your parents' home is also a problem. And this bleeding that happens unexpectedly and lasts for weeks also becomes bothersome.

Women in both countries believed that certain side effects were interconnected. For example, in 3 of 9 Rwandan FGDs and 7 of 10 Kenyan FGDs, some women associated amenorrhea with the possibility of swelling, weight gain, abscesses, infertility, or cancer. A Kenyan injectable user said:

When in my second year of using the injection, I completely stopped getting my periods. My stomach started to swell, and it reached a time instead of the period, I was very wet in my vagina and my sexual desire diminished.

On the other hand, not all side effects were viewed negatively. Several women in Rwanda and one in Kenya appreciated weight gain due to injectable use. Several dozen participants, including 6 Kenyan providers/program managers and potential users from both countries, associated amenorrhea with time and cost savings. One rural injectable user in Rwanda explained:

The first advantage [of the injectable] is that you don't see your period and so you don't spend money buying Kotex or soap to wash your sanitary napkins. You have that money for other things.

Women indicated that most contraceptive methods had side effects. Indeed, women's descriptions of side effects related to pills, injectables, and implants—and sometimes even intrauterine devices (IUDs)—were similar. They also indicated that side effects were frequently transitory. For many women, side effects were problematic only if they lasted a long time or were so severe that they disrupted women's normal routines. In such cases, they might assume that the contraceptive method being used did not suit their bodies, leading them to discontinue or switch methods.

**Side Effects and LAI Approaches:** A common concern about a potential LAI, particularly among women who had already experienced side effects with DMPA, was that it might double the intensity of side effects. In several FGDs and IDIs, participants suggested that developing a nonhormonal LAI would be preferable, although some equated such nonhormonal approaches to the IUD, which was also considered to cause side effects for many women. A current implant user from Rwanda reported:

Even the 3-month one [injectable] caused me serious bleeding and an excess of hormones above those already in my body. In my opinion, they should try to lower the quantity of hormones or just simply [make] a product without any hormones—and many would choose this method. Because when others see the effect that [the 3-month injectable] had on me, or on someone else who had the same problems I did, they are afraid to run the same risk.

Others believed that women's experiences with injectable contraception were variable and related to their body's own chemistry. Consequently, if a woman did not experience side effects from a current hormonal method, she would be unlikely to experience them with a longer-acting one, as expressed by one Rwandan injectable user:

You see, if the injectable was given every 6 months instead of every 3 … that would be very good for me. I also think there wouldn't be any side effects, because we don't have any with the 3-month injectable we are using.

### Return to Fertility: Similar to Women Who Have Stopped Using Nonhormonal Methods

In general, potential users were more concerned than providers about return to fertility, and this characteristic was of more concern in Kenya than in Rwanda. In considering an LAI, women preferred a product that provided an immediate return to fertility; however, a longer period of time—even as long as 18 months—could be acceptable, particularly if the time period were predictable. International opinion leaders generally agreed that return to fertility within a reasonable and predictable time frame was an important priority. One respondent stated:

*Return to fertility could be problematic if it extended far beyond what we already have with the 3-month injection*.

Women preferred an immediate return to fertility, but a longer time frame could be acceptable if it were predictable.

Another respondent noted that if an LAI were associated with a substantially longer return to fertility than DMPA, it “would be a major issue for women.” (Women who stop using DMPA become pregnant, on average, 10 months after their last DMPA injection.^16^)

**DMPA Experiences:** Many potential users and providers in both countries recounted stories about long delays in the return to fertility after injectable discontinuation. And, in more than half of Kenyan FGDs and one-third of Rwandan FGDs, some women believed that injectable contraception could lead to infertility. Delays were most commonly attributed to injectable-induced changes in menstruation—either heavy bleeding or amenorrhea—and frequently reflected fundamental misunderstandings about human anatomy and contraception. For example, a Rwandan woman associated heavy bleeding with fertility delays:

I know about a woman who was using the 3-month injectable and she was bleeding a lot, but later, when she wanted to have another baby and stopped using it, she waited a long time without getting pregnant—for at least 5 years. When she went to the doctor, she was told she would no longer be able to have children because the bleeding carried away her eggs—that little remained for her to conceive.

Women attributed delays in return to fertility to bleeding side effects of contraceptive methods.

A Rwandan provider suggested that long periods of amenorrhea could lead to delays:

We can't say this to our clients, but our doctor here always tells us to counsel women to do a sonogram at least once a year, because they continue to use the 3-month injectable for a long time without knowing if the uterus has atrophied. And when the time comes to want to get pregnant, she doesn't conceive because the uterus has atrophied … she begins to worry.

Most Kenyan providers agreed that delays in the return to fertility were a concern for women, but even more so for partners. And while few providers suggested that there might be a connection between injectable use and infertility, delays in the return to fertility made some reluctant to counsel nulliparous women about injectable use. As one Kenyan provider reported:

The injectable cannot make one sterile—not unless you had [just] one good ovum … we tell them not to start using family planning when one has not given birth, because a woman can start using family planning and they don't have ova to conceive. You know, she will blame family planning and yet she is the one with the problem, or maybe that is the way she has been created. So, it is good to start using family planning when one has a child … That is why it is referred to as family planning.

Nevertheless, some women—and even some providers and policy makers—believed that other contraceptive methods were likely to induce even longer delays in the return to fertility, suggesting that return to fertility was somehow directly related to a method's duration of protection. In Kenya, a policy maker reflected:

Mmm … I guess probably it's sometimes explained [from the perspective of] a provider in terms of a continuum … With the oral contraceptive pill it's immediate; with the injectable it'll take a period from you know, 1–3 months, or even up to 6 months with the implant … and so, I think that depending on how it is explained to the client, [it] may be a cause of anxiety.

**Return to Fertility and LAI Approaches:** Acceptability of a longer return to fertility for an LAI seemed to depend on women's—and men's—fertility desires. If couples were looking for longer protection, they would not care about the long return to fertility. Furthermore, if the delay in return to fertility were predictable, many suggested that a longer return to fertility might be perceived as a benefit. A Rwandan provider said:

If this method had a return to fertility after 18 months for everyone, all women would use it because [if she wants to get pregnant in 4 years] … she'll use the method for 1½ years and she'll wait another 1½ years even though she's [already] stopped the method. I assure you it's like this. Even those who use implants or the IUD will remove them to use this injectable. Even me, my wife doesn't use the injectable but if it's like this, I will be interested in this long-acting injectable.

Some, but not all, participants conflated the long contraceptive “tail” (the period in which the drug stops having an acceptable level of effectiveness but has not been fully eliminated from the body) with an extended period of effectiveness. Thus, some providers in both Kenya and Rwanda reasoned that:

Alternatively, you can calculate the time that you want to get pregnant and stop like a year before the time you wish to get pregnant. If you wanted to get pregnant after 3 years, you could use the new method for 2 years and stop, then you will get pregnant after 1 year, which will add up to 3 years.

Reacting to a similar argument in her discussion group, one Rwandan woman exclaimed:

I was thinking about what my colleague just said. When she just said that she'd stop the injectable when her child is 4 years old, is she sure that this injectable will be 100% [effective], so that when she stops, she'll spend 18 months without getting pregnant? Why do you put it in that kind of logic? With the injectable we normally use, you can stop and fall pregnant right away. Why don't you think it could be the same thing, remembering that it [fertility] also depends on each one's body?

Finally, several providers suggested that it would be very useful to identify a way to reverse the effect of the drug, so that women whose fertility intentions changed would be able to get pregnant more quickly. In Kenya, a provider proposed:

If there could be a way one would get an injection or drug to reverse back the medication any time they want a baby, instead of waiting for the whole period the drug would last, then that would be fine, like for instance the way one gets rid of pills and conceives almost immediately, or getting rid of [an] IUD.

A handful of potential users from different Kenyan FGDs also raised the possibility of manufacturing an antidote. As described by one non-injectable user:

*So they have to go and look for this* miti ni dawa *[herbal treatment] so that they can go and wash their stomach … That is a traditional drink … herbs … that you take so that it can go and wash … those chemicals which is there.*

### Delivery System: Single-Dose, Prepackaged, Disposable Injection System

Potential LAI approaches under consideration could result in changes to product presentation. For example, while the TPP aims for a single injection, increasing the dose of an existing formulation might require co-administration of 2 doses, whereas changes in the drug delivery system could require a 2-vial system.

For women in about one-third of the FGDs—mostly in Rwanda—characteristics of product presentation were important to consider for a new LAI. Providers were more divided on whether such characteristics were important. A few women in FGDs from more rural community-based distribution (CBD) programs felt 2 injections were not problematic, but most women and providers strongly preferred an LAI to be in a single, prepackaged injection. Kenyan providers worried about client acceptability, especially with regard to pain or discomfort. In both countries, a few women and providers added that providers were not always well-trained, so 2 injections could lead to even more swelling and pain. For example, a Kenyan provider commented:

Considering that human beings fear pain, it [2 shots] would minimize the number of clients and so you can be sure that most women would lose interest in the injectable.

A single, prepackaged delivery system was generally preferred to facilitate provider provision and reduce user pain or discomfort.

Similarly, a Rwandan injectable client from a private NGO clinic said:

It's best to have 1 injection instead of 2 because some people swell at the site of injection. For example, me, after an injection I have pain for about a week. So, it would be preferable to swell at 1 site rather than 2 sites.

For some Rwandan women, receiving 2 shots instead of 1 also increased the perception that one was receiving a very high dose of hormones. A woman from a public hospital setting in Kigali spoke for her fellow participants when she said:

Why have we all said, “No?” It's because we are worrying about the consequences. I told you that I had an injection and I bled from the 1st to the 30th. And I imagine that the hormones will be double the quantity; I won't be able to continue [like that] for 6 months. In that case, if you were using sanitary napkins, you would have to look for Pampers. … Instead of giving you 2 injections at the same time, I wish that they could mix the 2 medicines and give them in a single injection.

Providers and potential users in both countries were also concerned about the idea of having to mix medicines before giving an injection. Concerns related to improper mixing, and consequently a reduction in safety or effectiveness. Participants raised the same kinds of concerns if multiple injections were given from a single vial. For example, one injectable user in Rwanda worried that the medicines from the 2 different injections might not “meet” (within her body) causing her some concern about the effectiveness of the LAI. Others described having more confidence in a product that was already premixed and packaged; it would be possible to check the expiry date as well. A potential user from Rwanda explained:

I think it would be a good thing if the longer-acting injectable is put in a single dose, because it would prevent people from having to first mix it before giving it, or from not drawing out all of the medicine from the vial. The provider will spend less time with the client, because he is not going to have to prepare. It will also prevent him from giving an incomplete dose to the clients.

Finally, current or past injectable users in 2 of the Rwandan FGDs, as well as a dozen providers from Kenya and Rwanda, preferred that a new LAI be delivered through disposable needles to “protect users from infections.”

### Cost: US$4 or Less Per Year in a Public-Sector Program

Cost was one of the most important considerations for international opinion leaders, policy makers, and providers, but one of the least important considerations for most potential users. For the opinion leaders, affordability was the second most common response (14 of 28) to an open-ended question about which characteristics would be most important for a new LAI. One opinion leader stated:

*Cost of the final product may be a challenge. It should be less than the cost of 2 DMPA 3-month injections we currently use*.

Cost was an important consideration for policy makers and providers but not so much for potential users.

Another respondent said that a key issue is:

*Price … [An LAI] needs to compete; I'd say a unit price at less than [US]$4 per year*.

The reasons for women's low concern about cost were varied. Women in some Kenyan FGDs were already paying for contraception and consequently appeared willing to pay US$4, or approximately 350 shillings, per year for an LAI. For example, women already paid 20–100 shillings (US$0.24–$1.18) per cycle of pills, and some IUD or implant users reported paying 1,500–3,500 shillings. And, although injectables were free through the public sector, Kenyan women who obtained their method through a private-sector clinic or were compelled to buy their injectable at a chemist or drug shop because of stockouts, reported paying 150–300 shillings per injection. Clinics catering to higher-income segments might charge even more, according to several Kenyan policy makers and program implementers.

In Rwanda, a few women who received their injectable at a private or NGO facility reported paying 700–1,000 francs (US$1.16–$1.66) for a 3-month injectable, and several women suggested that the 2-month injectable cost 1,500 francs per dose, or 9,000 francs per year. Nevertheless, most women reported that they would not pay US$4 (approximately 2,400 francs) for an LAI, given that all other methods could be obtained for free. Women in the more rural FGDs were especially clear about the difficulty of paying. For example, one woman said:

It will be used by women with money. I'm not saying it is not going to be used, but we have a lot of poor people. There are some who may not even have 100 Rwandan francs a month. Others might have that, but they have a family. You know, child care may cost 5,000 or 6,000 francs a month.

On the other hand, women in several FGDs per country suggested that injectables can lead to cost savings, especially due to reduced bleeding and therefore less need to purchase hygiene products. Several also suggested that fewer trips to the clinic for an LAI would save money. Policy makers and providers also added that there could be systems savings due to lower client load.

## DISCUSSION

This research identified strong support for the development of a longer-acting injectable contraceptive method for multiple reasons. An LAI would build on the existing popularity and high use of injectables; reduce the travel time and number of clinic visits needed for users; and increase convenience.

Some of the findings about desired attributes have immediate implications for LAI development activities. For example, there was little disagreement that a new LAI should be **highly effective**. The actual level of effectiveness needed to generate demand may vary somewhat, but potential users and providers thought the LAI would be acceptable as long as the method is perceived to be as effective as current injectable formulations. Additionally, potential users and providers expressed strong preferences for a product that was delivered in a **single injection** rather than 2 injections, was **prepackaged**, and could be **disposed** after one use. A prepackaged, single use, disposable LAI was associated with less pain for injectable users, higher levels of product safety and efficacy, and greater efficiency for providers.

A potential longer-acting injectable should be highly effective and delivered in a single, prepackaged, disposable system.

Other TPP-related information indicates the need for more tailored communication and counseling approaches to ensure acceptability and adherence within clinical trials and beyond. For example, while almost all participants agreed that an LAI with **few or no side effects** and a **rapid return to fertility** was desirable, they were also quick to recognize that most contraceptive methods had side effects. Some side effects—especially heavier bleeding or loss of libido—were viewed as less acceptable than others. If LAI use were associated with lengthy periods of such side effects, it would offset one of the important perceived benefits of injectable use—that of discretion or privacy—by drawing attention to contraceptive use and potential disapproval from non-supportive partners. Other side effects, such as amenorrhea, appeared tolerable or even appreciated, as long as users and their providers had correct information about them. Similarly, although different lengths of return to fertility might affect the types of women most likely to use an LAI, many participants anticipated that an 18-month return to fertility could be acceptable, if women were properly counseled about it.

Lengthy menstrual bleeding as a potential side effect of a longer-acting injectable would offset the benefit of discreet use of injectables.

The study did identify widespread misunderstanding about contraception, in general, and injectables specifically, among both providers and users. In particular, misperceptions about the effect of menstrual side effects on fertility, as well as variation in and reasons for injectable-related return to fertility should be addressed for existing injectables and new LAI methods.

Finally, this assessment of TPP characteristics revealed some interesting country-level differences, serving as a reminder that product demand is likely to depend on the specific context. In Rwanda, the potential for an LAI to last 6 months was a welcome alternative to shorter-acting methods such as condoms, pills, and 3-month injectables, particularly in more rural areas where contraceptive resupply could be difficult. In Kenya, where women appeared to access a wide variety of contraceptive options at a range of costs through a more diverse set of public, private, and/or NGO clinics, cost and the potential for service provision through community-based workers were less important, while concerns about ensuring the quality and control of LAI delivery were greater.

### Limitations

The study has several limitations. We tried to reduce selection bias by approaching potential participants directly, rather than having clinic staff identify and recruit them. Nevertheless, because participants were drawn from health facilities, our findings may not represent the opinions of women who do not access family planning services through clinics or who do not use contraception at all. The study's qualitative design and small sample size prevent us from making recommendations about the composition of specific characteristics that would optimize demand. However, the in-depth discussions about TPP characteristics from a range of stakeholders including policy makers, program managers, providers, and potential LAI users offer evidence of widespread support for the introduction of an LAI, as well as guidance about product characteristics that may be most or least important to target. Further quantitative research would be needed to determine the extent to which themes identified in this study can be generalized.

## CONCLUSION

This study provides evidence of strong acceptability for an LAI. Furthermore, it provides some guidance related to product characteristics that should be prioritized in the development process, while also serving as a reminder that eventual demand will be influenced by policy and service delivery decisions that affect potential users' knowledge about, access to, and correct use of the method.
